# Special Issue “Membrane Technologies for Sustainable Biofood Production Lines”

**DOI:** 10.3390/membranes11070485

**Published:** 2021-06-29

**Authors:** Laurent Bazinet

**Affiliations:** Department of Food Sciences, Laboratoire de Transformation Alimentaire et Procédés ElectroMembranaires (LTAPEM, Laboratory of Food Processing and ElectroMembrane Processes), Dairy Research Center (STELA), Institute of Nutrition and Functional Foods (INAF), Université Laval, Québec, QC G1V 0A6, Canada; laurent.bazinet@fsaa.ulaval.ca; Tel.: +1-418-656-2131 (ext. 407445); Fax: +1-418-656-3353

Population growth and urbanization present serious challenges for the biofood sectors since there will be a 70% increase in the global demand by 2050. To satisfy these demands, the food, biotechnological, and biopharmaceutical industries should significantly increase their productivity. However, these industries are aware that in addition to controlling their impacts on the environment, they will have to maintain their levels of safety and quality standards. In this context, the aim of the Special Issue is to obtain a holistic picture of the latest advances in membrane technologies oriented towards the improvement of the biofood production line sustainability.

Consequently, this Special Issue of Membranes provides an updated and comprehensive overview of the state of fundamental knowledge on membrane phenomena, electrodialysis market, fouling mechanisms, and new membrane applications in the biofood industry, combining original experimental and modeling works, as well as reviews, prepared by renowned experts. The articles cover the following highlights: (1) Improvement of filtration performances, (2) Fouling identification, mechanisms, and their mitigation, (3) Production of healthier or bioactive products, (4) Prediction of bioactive peptide separation performances, (5) Recent developments in membrane processes, and (6) Use of electrodialysis processes in sustainable strategies. A brief descriptive summary of the scientific contributions is reported here.

In the paper of Perreault et al. [1], the filtration performances of cranberry juice for its clarification were improved by use of pectinolyticenzyme. In terms of industrial application, a 60 min depectinization coupled to a clarification step by a 500 kDa UF membrane were found as a good compromise between the enhancement of filtration performance, the loss of polyphenols (44–58%), and their fouling at the membrane surface. During milk protein fractionation by a 0.1 µm microfiltration (MF) of pasteurized skim milk, Schiffer and Kulozik [2] determined, on a membrane system performed in a mode close to an industrial plants’ operational mode, the maximum possible filtration time per filtration cycle and the cumulated number of operational hours per year as a function of the processing temperature. The main stopping criteria highlighted by the authors were the microbial count (max. 10^5^ cfu/mL) and the slope of pH change as a function of filtration duration. The conclusions of this paper can help to minimize the use of cleaning agents by reducing the frequency of cleaning cycles and consequently to maximize the active production time.

In their papers, Perreault et al. [1], Ge et al. [3], Wang et al. [4], and Abou Diab et al. [5], reported the fouling of membranes by respectively polyphenols and anthocyanins (UF membrane), charged organic anions (anion exchange membranes), and divalent ions (cation exchange membrane), amino acids, and peptide or hem-peptide (cation-exchange membrane). Concerning the mechanisms, the results of the study by Ge et al. [3] indicated that the membrane fouling is caused by two different mechanisms: electrostatic interactions of charged organic anions with the anion exchange membranes and precipitation of Ca^2+^ and Mg^2+^on the cation exchange membrane, by reaction with OH^−^ ions generated by water dissociation. The chemical cleaning with alkaline and acid was proposed to mitigate these fouling and scaling [3]. In their work, on the production of hydrolysate from bovine hemoglobin by electrodialysis with bipolar membrane (EDBM), Abou Diab et al. [5] explained the fouling of perm-selective cation exchange by the fact that, according to the pH of the solution and the release of hem alone or protein fragments containing hem and peptides during hydrolysis, peptides may have first interacted electrostatically (positive residues) into the scattered cavities of the thin charged layer (forming the perm-selective layer). Indeed, into these scattered cavities, the negatively fixed groups of the cationic matrix were reachable and, afterwards, hem or the protein fragments containing hem interacted with these already adsorbed peptides via hydrophobic interactions. These results confirmed the hypothesis developed by Persico and Bazinet [6] for a perm-selective cationic membrane and a whey protein hydrolysate. Additionally, Abou Diab et al. [5] proposed to minimize the fouling by applying pulsed electric field or an overlimiting current regime, by changing the configuration or by modification of ion-exchange membranes. The main avenues to minimize foulings and scalings by different species present in water or food fluids and the mechanims and ways to mitigate them are presented in detail in the paper of Bazinet and Geoffroy [7].

Concerning the production of healthier products, Wang et al. [4] used conventional electrodialysis to demineralize soy sauce. Indeed, soy sauce is a common condiment with a unique flavor derived from its rich amino acid and salt contents. However, excessive intake of high-sodium food affects human health by leading to hypertension and kidney disease. The optimal ED desalination conditions were at a current density of 5 mA/cm^2^ and pH of 5, with a 64% desalination rate, a 29.8% amino acid loss rate, and that without compromising the original flavor. With the goal of producing a bioactive hydrolysate, from bovine cruor, a slaughterhouse waste, Abou Diab et al. [5,8], developed a new membrane-based hydrolysis process. In these studies, EDBM was demonstrated for the first time as an ecoefficient and innovative technology to produce peptide hydrolysates from enzymatic hydrolysis of bovine hemoglobin with low mineral salt concentration. This was due to the specific characteristics of the bipolar membranes which allowed to generate in situ H^+^ and OH^−^ ions from water dissociation under an electrical field, and consequently do not need the use of any chemical agents. Hydrolysates obtained by EDBM showed excellent antimicrobial and antioxidant activities, as well as, for the first time, anti-fungal activities. Hydrolysis in EDBM showed the same enzymatic mechanism, “Zipper”, as observed in conventional hydrolysis and allowed the generation of 17 identified bioactive peptides such as neokyotorphin (NKT, α137-141).

Since neokyotorphin (NKT) released from the hydrolysis of hemoglobin presents a major interest for food safety, Beaubier et al. [9] worked on the concentration of NKT and its separation performances by UF. A simulation method for predicting the NKT yield and enrichment by UF was studied on colored and discolored bovine hemoglobin hydrolysates at different degrees of hydrolysis with regenerated cellulose membranes. A remarkably high experimental NKT purity of about 70%, corresponding to an enrichment factor of about 29, compared to the initial hydrolysate, was obtained for the ultrafiltration of the colored 3% hydrolysis degree hydrolysate with a 1 kg·mol^−1^ MWCO-regenerated cellulose membrane at a VRF of 5. The results showed that the antimicrobial activity was in relation to the process selectivity and NKT purity. This reliable method of prediction is part of a global approach to valorize protein resources in a rational way from various by-products.

Amongst the recent developments, Talebi et al. [10] proposed the use of reverse osmosis or electrodialysis for recovering lactic acid and adding value to the acid whey treatment process. Indeed, acid whey is the principal co-product of a wide variety of dairy products, such as fresh cheese, caseinate, and Greek yogurt, and its production is increasing every year to answer the actual popularity rise of these products. Consequently, its valorization represents a great deal for the dairy industry around the world. In their papers, the authors reported a partial separation between lactic acid and potassium chloride by reverse osmosis and electrodialysis. Neither process was able to achieve sufficient separation to avoid the use of further purification steps. This indicates that the separation of lactic acid from complex feed solution is still a challenge. Other recent developments were presented in the reviews of Vollet-Marson et al. [11] and Bazinet et Geoffroy [7]. Hence, Vollet-Marson [11] emphasized the recovery of valuable peptides from hydrolysis of spent yeasts, the second most generated by-product from the brewing industry. They first focused on the current strategies, challenges, and solutions for the application of conventional pressure-driven membrane technologies to the downstream processing of protein hydrolysates. Then, they presented the use of charge-based membrane separation techniques such as high-performance tangential flow filtration (HPTFF), electrophoretic membrane contactor processes, membrane chromatography, electrically enhanced membrane filtration (EMF), UF using charged membranes, electro-ultrafiltration using pulsed fields, electrodialysis and electrodialysis with UF membranes (EDUF, or generally speaking, electrodialysis with filtration membrane (EDFM)). According to these authors, among them, one of the most promising techniques recently used for peptide separation is electrodialysis and EDUF. They also discussed the particularities involving the separation of spent brewer’s yeast protein hydrolysates by taking into account engineering, technical, and practical aspects of membrane processes. In addition, in a context of preserving and improving human health, Bazinet and Geoffroy [7] confirmed that electrodialytic processes are very promising perspectives. Indeed, these processes allow the treatment of water, preservation of food products, production of bioactive compounds having health benefits, extraction of organic acids, and recovery of energy from natural and waste waters with minimal environmental impact. Hence, the most recent developments based on electrodialytic membrane phenomena such as applications of electroconvective vortices, use of pulsed-electric field, electrodeionization and shock electrodialysis are discussed. Additionally, the authors provided a global overview of the actual electrodialytic equipment market and manufacturers.

Finally, the use of electrodialysis processes in sustainable strategies or in the framework of a circular economy are presented. The circular economy is an economy that operates in a loop, consequently avoiding the notion of waste and its objective is to produce goods while strongly limiting the consumption of raw materials and non-renewable energy sources. Hence, in their review, Bazinet and Geoffroy [7] give a global portrait of the most recent uses or potential uses of electrodialytic membrane processes in sustainable strategies. For this purpose, the authors mainly focused on articles reported in the literature from 2015 to June 2020. Based on these new developments or technologies, the authors presented and proposed the integration of electrodialytic technologies in sustainable strategies or use of eco-efficient new electrodialytic technologies such as electrodialysis with filtration membrane (EDFM). It appeared that new knowledge on pulsed electric fields, electroconvective vortices, overlimiting conditions, and reversal modes, as well as recent demonstrations of their applications, are currently boosting the interest for electrodialytic processes. However, the hurdles are still high when dealing with scale-ups and real-life conditions. In their papers, Abou Diab et al. [5,8] developed and proposed a new green application of EDBM to the production of bioactive peptides from a by-product of slaughterhouses, which fits perfectly with the concept of a circular economy. Indeed, hemoglobin from blood, once hydrolyzed, allows the bioproduction of active peptides that can then be reused on meat or meat products to increase their preservation and inocuity.

The increasing need of more sustainable and performant processes for biofood product transformation or by-product valorization is strongly pushing towards the development of (1) fundamental knowledge to improve the processes, modify the final products or avoid fouling, (2) new applications to add value to the treated product or modify its biological activity, and (3) new technological concepts such as electrodialysis with filtration membrane (EDFM). However, due to the intrinsic nature of the biofood products or by-products, fouling and long-term maintenance of the membranes still remains a challenge to reduce costs and limit fouling for better performances. Furthermore, as demonstrated in some papers, under the concept of circular economy, an emerging concept, the by-products, wastes from the biofood industry, can be recycled into raw materials of the same or another industry for production of bioproducts with improved functions. In the frame of this scenario, the present Special Issue aims to offer an overview about the different applications and strategies available to achieve sustainable biofood production lines, in particular in the field of pressure-driven and electrically-driven membrane processes.

## Data Availability

Not applicable.
